# Semi-automatic Extraction of Functional Dynamic Networks Describing Patient's Epileptic Seizures

**DOI:** 10.3389/fneur.2020.579725

**Published:** 2020-12-11

**Authors:** Gaëtan Frusque, Pierre Borgnat, Paulo Gonçalves, Julien Jung

**Affiliations:** ^1^Univ Lyon, Inria, CNRS, ENS de Lyon, UCB Lyon 1, LIP UMR 5668, Lyon, France; ^2^Univ Lyon, CNRS, ENS de Lyon, UCB Lyon 1, Laboratoire de Physique, UMR 5672, Lyon, France; ^3^National Institute of Health and Medical Research U1028/National Center for Scientific Research, Mixed Unit of Research 5292, Lyon Neuroscience Research Center, Lyon, France; ^4^Department of Functional Neurology and Epileptology, Member of the ERN EpiCARE Lyon University Hospital and Lyon 1 University, Lyon, France

**Keywords:** epilepsy, functional connectivity, SEEG, epileptogenic networks, dynamical graph, subgraphs extraction

## Abstract

Intracranial electroencephalography (EEG) studies using stereotactic EEG (SEEG) have shown that during seizures, epileptic activity spreads across several anatomical regions from the seizure onset zone toward remote brain areas. A full and objective characterization of this patient-specific time-varying network is crucial for optimal surgical treatment. Functional connectivity (FC) analysis of SEEG signals recorded during seizures enables to describe the statistical relations between all pairs of recorded signals. However, extracting meaningful information from those large datasets is time consuming and requires high expertise. In the present study, we first propose a novel method named Brain-wide Time-varying Network Decomposition (BTND) to characterize the dynamic epileptogenic networks activated during seizures in individual patients recorded with SEEG electrodes. The method provides a number of pathological FC subgraphs with their temporal course of activation. The method can be applied to several seizures of the patient to extract reproducible subgraphs. Second, we compare the activated subgraphs obtained by the BTND method with visual interpretation of SEEG signals recorded in 27 seizures from nine different patients. As a whole, we found that activated subgraphs corresponded to brain regions involved during the course of the seizures and their time course was highly consistent with classical visual interpretation. We believe that the proposed method can complement the visual analysis of SEEG signals recorded during seizures by highlighting and characterizing the most significant parts of epileptic networks with their activation dynamics.

## 1. Introduction

About 30–40% of epileptic patients are drug resistant (1). For those patients, surgical resection of the epileptogenic brain structures is considered to promote seizure freedom (1). Intracranial EEG using depth EEG recordings or the stereoencephalography (SEEG) method is often required to guide tailored-surgical resection (2, 3).

The primary aim of SEEG is to delineate precisely the epileptogenic regions. However, since the pioneering works in SEEG, it has been shown that seizures cannot be considered as static phenomena with a single focus activation leading to clinical manifestations (4). SEEG recordings of focal seizures typically show that the epileptic activity spreads during seizures across several anatomical regions. It begins at the seizure onset zone and spreads toward remote brain areas. This dynamical pathological process can be described by several brain states characterized by transient and abnormal connectivity profiles within the epileptogenic network (5). A full and objective characterization of this patient-specific dynamic network is crucial for optimal surgical treatment.

Understanding brain network modifications operating at different time scales in the interictal state and during seizures is a very active stream of research. This is in line with computational neuroscience studies modeling neurological diseases as brain network disease (6–11).

In the field of epileptology, the structure of epileptic (or ictal) networks has been explored using connectivity analysis (functional or effective connectivity) and more recently, graph-theory analysis, (5). Functional connectivity (FC) approaches, based on linear or non-linear measures, quantify the statistical relations between any pair of recorded SEEG signals and their evolution across time. In most of the studies, pairwise correlations between remote sites are computed at specific time points [for a review, see (5)]. Those approaches unveil the organizational interactions of different regions of interest in the brains. They have proved very fruitful to investigate the neurophysiological correlations of symptoms during seizures, or to describe several subtypes of focal epilepsy involving specific networks (12–14). Graph theory is the study of graphs, which are mathematical structures used to model networks, and specifically pairwise relations between objects (8, 15). A graph is made up of vertices (also called nodes) that are connected by edges. Graph theory approaches allow the description of both local and global characteristics (16). For epilepsy, the nodes usually represent electrode contacts and the edges represent the FC measures. The resulting graph structure is known to contain relevant fingerprints of the seizure dynamic. Studying network structures with graph theory provides mathematical tools to investigate different subtypes of epilepsy (17). For example, it has been shown that the properties of networks' topology are different for temporal lobe, mesial temporal lobe, and neocortical epilepsy (18). Moreover, some studies suggest that investigating local properties of the network structure through graph theory concepts provides biomarkers for epileptogenic focus localization (19–21). Lastly, dynamic graph theory can also provide step-by-step modeling of the propagation of the seizure in the brain (19, 22).

A major challenge for the study of ictal networks is that seizures are a highly dynamical process with rapid transitions between network states (5, 23). To track network changes during the seizures, the network organization has to be described at short time scales. In the most straightforward approach, FC approaches forming the backbone of the network are estimated at different time steps of the seizure. Therefore, they are prone to create spurious connections and constitute a noisy estimation of the pathologic dynamic network. Moreover, as the brain activity is commonly monitored with more than 100 electrode contacts distributed along the stereotactic rods, the number of FC scales quadratically (100 electrode contacts providing around 5000 FC measures), making the study of FC over time a resources consuming task that requires high expertise.

One advantage of SEEG monitoring is to allow the recording of several seizures from the same patient, with the same measurement points (or nodes), yielding thus as many realizations of identically distributed dynamical networks of FC. Hence, several seizures from the same patient can be investigated that may share some connectivity features but with different dynamics. Methodological tools have to be proposed to extract relevant information from those large datasets. Ideally, such methods should summarize the dynamics of the seizures by providing several epileptogenic networks, with stable network structures, that characterize the different steps of the seizures and that are involved in the production and the propagation of the ictal events. Those network states may be common to the different seizures of the patient, but the timeline activation should follow a pattern that remains specific to each seizure (22).

Along these lines, we propose a novel semi-automatic method to characterize the dynamic epileptogenic network quantitatively and across time using SEEG signals. We propose to perform the joint analysis of all seizures of the same patient, first to reduce the measurement uncertainty in the calculation of FC indices, and second to make robust the identification of a time-functional pattern, systematic in all seizures and characteristic of a patient's pathology. First, the full FC matrix is computed for each time step using a classical FC measure, namely the Phase Locking Value (PLV) (24, 25). Then, the method extracts several pathological subgraphs with their own activation score during seizures. We expect each subgraph to comprise several brain nodes with high connectivity values. The paper is organized as follows: We extend our previous work (26) to seizures with different durations, we call this method the Brain-wide Time-varying Network Decomposition (BTND). On the application side, we validate the clinical use of the method on a larger clinical dataset.

## 2. Materials and Methods

### 2.1. Description of the Brain-Wide Time-Varying Network Decomposition Method

**Figure 1:**
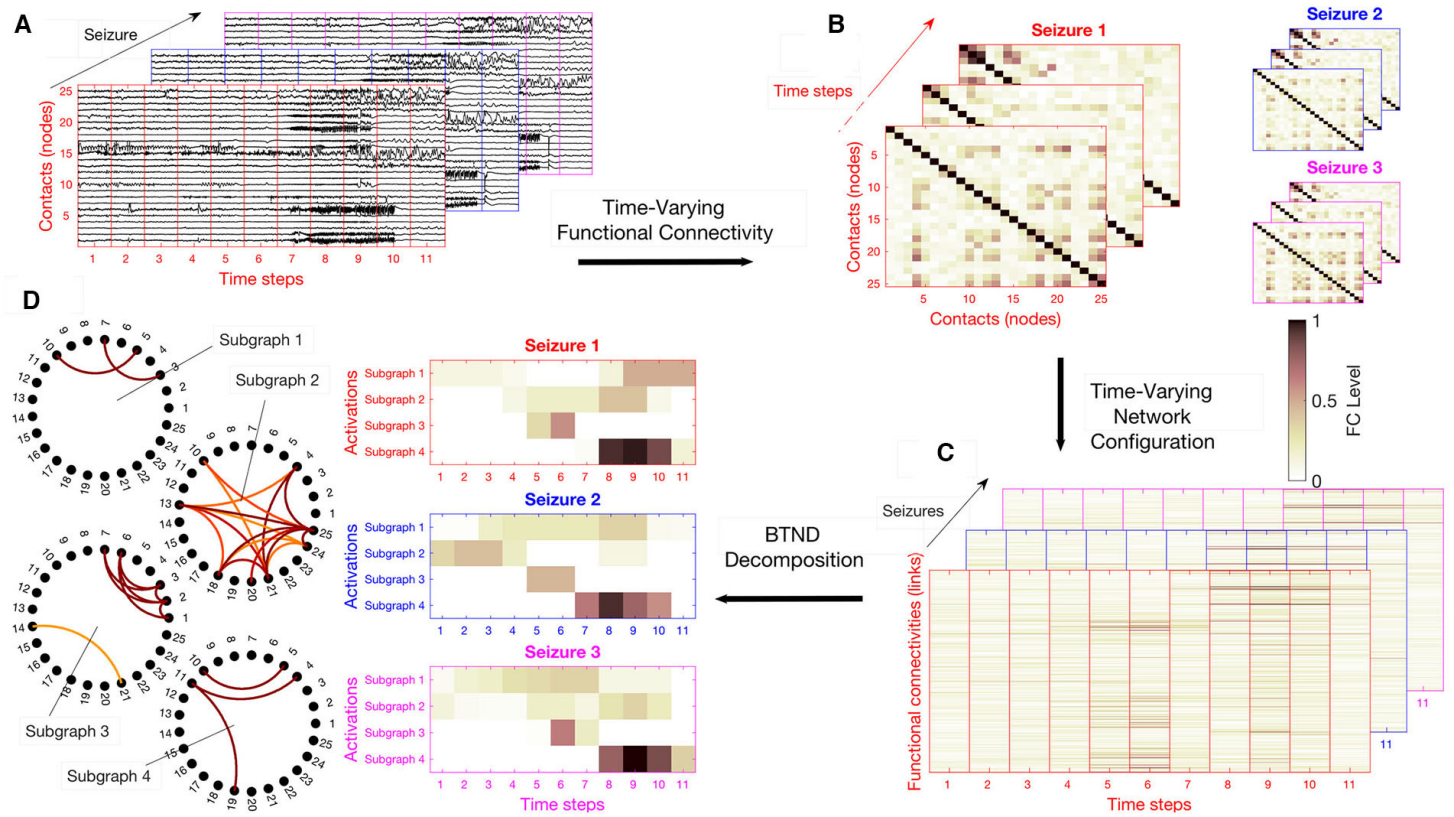
Overview of the strategy: **(A)** We chop each recording in time segment, potentially with some overlapping. **(B)** For each segment, we compute the connectivity level of each pair of electrode contacts with a Functional connectivity (FC) measure. **(C)** We rearrange the FC measures in a vector, stacked in time they form for each seizure an FC matrix. **(D)** The list of FC matrix is decomposed into a set of FC graphs with their activation profiles respective to each seizure. Here, the number of subgraph is *K* = 4, and the activation profiles of each subgraph are represented for the 3 available seizures of this patient.

For each patient, the dataset is composed of several SEEG recordings for different seizures. Seizures can have different durations. The proposed strategy can be summarized in four steps: (a) We chop each recording into short segments; (b) for each segment, we estimate via FC measures the connectivity for each pair of electrode contacts; (c) we rearrange the FC measures into a list of matrices representing the time evolution of FC for each seizure of a patient; (d) the list of matrices representing the multi-seizures brain-wide time-varying network is decomposed into FC subgraphs characteristic of one patient but common to all his seizures, along with their activation profile specific to each seizure. The main steps of the method are illustrated in Figure 1.

### 2.2. Representation of the Multi-Seizure Brain-Wide Time-Varying Network

Practically, FC measurements are stored in a three-dimensional structure **X**_*lt*_{*s*} corresponding to the FC of index *l* at time segment *t* and for the seizure *s*. The list of matrix **X**{*s*} ∈ ℝ^*L*×*T*(*s*)^ ∀*s* (1, ..., *S*) is the mathematical representation of the multi-seizures brain-wide time-varying network. Here, *S* is the total number of seizures recorded for the same patient. We remind that the number of time steps *T*(*s*) can vary for each seizure. Figure 1C illustrates an example of list of matrices **X**{*s*}.

### 2.3. The Optimization Problem Related to the BTND Method

The core of the BTND method is then to seek, for each seizure, for the following decomposition:

(1) $$\begin{array}{r} \text{X}\left\{s\right\}\approx \text{F}{\text{V}}^{t}\left\{s\right\} \end{array}$$

where **F** ∈ ℝ^*L*×*K*^ contains the *K* FC subgraphs and **V**{*s*} ∈ ℝ^*T*(*s*)×*K*^ are their respective temporal activations corresponding to the specific seizure *s*. This approach directly entails the requested decomposition since the columns of the matrix **F** contains the weights of the edges in the sub-graphs, as it can be seen in Figure 1D and matrices **V**{*s*} directly correspond to the activation profiles of each seizure depicted in Figure 1D.

However, the solution for the decomposition (1) is not unique, and to favor handily interpretability from the medical viewpoint, we impose several constraints on the components **F** and **V**{*s*}:

- because most of the functional connectivity measures and activation indices are naturally positive values, we impose **F** and **V** to be **non-negative** matrices;
- to limit the complexity of the inferred subgraphs, we restrict the number of non-zero significant FC values, yielding a sparse matrix **F**. The **sparsity** constraint is meaningful in the context of epilepsy, where a large number of functional connectivities can be passively implied in the neurological process;
- to promote FC subgraphs that are continuously activated over specific periods, we impose **sparsity** and **compactness** on **V**{*s*}. These two constraints drastically improve the interpretability of the solution, as they prompt sparse and piecewise continuous activation periods that are close to cluster-like solutions. Formally, they correspond to the fused lasso constraint that reads:
  (2) $$\begin{array}{r} C\left(\text{v},\gamma ,\eta \right)\;:\;\gamma \overset{T}{\underset{t=1}{\sum }}|v_{t}|+\eta \overset{T-1}{\underset{t=1}{\sum }}|v_{t+1}-v_{t}|+\overset{T}{\underset{t=1}{\sum }}v_{t}^{2}\leq 1, \end{array}$$
  where the parameter γ and the parameter η compel each activation profile **v** = **V**_:*k*_{*s*}, to be sparse and compact, respectively. As for the third term in expression (2), it prevents incoherent solutions due to scaling indeterminacy (27).

Finally, the BTND boils down to an instance of joint non-negative matrix factorization (28–31) that takes on the following form:

(3) $$\begin{array}{r} \underset{\text{F},\text{V}\left\{1\right\},...,\text{V}\left\{S\right\}}{\text{argmin}}\overset{S}{\underset{s=1}{\sum }}\zeta_{s}{\left\|\text{X}\left\{s\right\}-\text{F}\text{V}{\left\{s\right\}}^{t}\right\|}_{F}^{2}+\lambda S\overset{K}{\underset{k=1}{\sum }}\overset{L}{\underset{l=1}{\sum }}|F_{lk}|,\\ \;s.t.\;C\left({\text{V}}_{:k}\left\{s\right\},\gamma_{s},\eta_{s}\right)\;\forall k\in \left\{1,...,K\right\},\;\forall s\in \left\{1,...,S\right\},\\ \;s.t.\;\text{F}\geq 0,\text{V}\left\{s\right\}\geq 0\;\forall s\in \left\{1,...,S\right\}. \end{array}$$

The ζ_*s*_ are free parameters to balance the relative importance of each seizure (in our study, we consider all seizures evenly and select the ζ_*s*_ parameter according to the energy of each seizure, see the Supplementary Materials for more precision). The sparsity factor γ_*s*_ and the compactness factor η_*s*_ are chosen to adapt to the particular duration of each seizure *s*.

Then, the method is associated with three hyperparameters: λ and γ, respectively, controlling the **sparsity** level of the FC subgraphs and the activation profiles, and η regulating the temporal **compactness** of the activation profiles.

### 2.4. Additional Comments on BTND

Let us stress that the optimization problem of Equation (3) is non-convex, and therefore, different initial conditions yield different solutions corresponding to local maxima. As it is common practice for non-convex methods in machine learning [e.g., *k*-means for data clustering, (32)], we repeatedly solve (3) with a different initialization and retain the one that reached the smaller minimum cost function. In practice, we empirically chose 20 trials. As for the other hyper-parameters, we observed that η = 0.2 produces coherent activation profiles. Also, fixing λ = γ simplifies the procedure without altering the results significantly. Then, λ is tuned so as to identify the 20% most resilient activated FC. To select the most pertinent number of subgraphs, we successively compute the decomposition for *K* ranging from 3 to 10. Based on an Elbow criterion and on visual inspection, we compare the quality of the resulting temporal activation and subgraphs, identifying thus the best value for *K*. Finally, we normalize each subgraph such that their connectivity strengths are between 0 and 1. We only retain connections above some threshold (empirically set to 0.2 in our experiments) to eliminate non-significant interactions.

For more details on the practical use of the method, see the Supplementary Materials. We provide the URL^1^ for a Github repository with Matlab implementation of the proposed BTND method.

## 3. Application on a Real Dataset of Epileptic Patients

### 3.1. Patients

To illustrate the clinical relevance of the BTND method, we applied the method on seizures recorded with intracranial EEG in 9 epileptic patients.

We included 9 adult patients suffering from drug-resistant focal epilepsy, followed in the Department of Functional Neurology and Epileptology at Lyon's University Hospital, who underwent intracranial EEG with SEEG according to the following criteria: (i) at least 1 seizure recorded during long-term monitoring; and (ii) conventional visual analysis of the SEEG signals identified clearly the seizure-onset zone.

Clinical details of all the patient included are listed in Table 1.

**Table 1:** Clinical details of each patient.

**Patient**	**Brain MRI**	**Interictal EEG**	**Ictal EEG**	**FDG PET**	**Ictal semiology**	**Surgery**	**Surgical outcome**
1	LT pole atrophy	LT slow wave activity and temporal spikes	LT ictal activity	left medial temporal hypoM + left anterior temporal neocortical hypoM	Oro alimentary automatisms + right hand dystonia + loss of consciousness	Left ALT	la (24 m)
2	LT post surgical sequelae	LT&RT spikes with left predominance	LT ictal activity	Left medial temporal hypoM + right medial temporal hypoM	Loss of consciousness + speech arrest	NA	NA
3	Right HS	Temporal spikes + temporal background slowing	RT basal ictal activity	Right medial temporal + RT pole hypo M + RT lateral neocortical hypoM	Left hand paresthesias + Ascending visceral sensation + tachycardia + loss of consciousness post icttal confusion	Right ATL	Ia (16 m)
4	Left HS, LT pole atrophy	LT&RT spikes with left predominance	LT ictal activity	LT pole + LT lateral neocortical hypo M	Oro alimentary automatismes + mental slowing with preserved consciousness	left ATL	la (48 m)
5	Post surgical right temporal lesion	RT spikes	RT ictal activity	RT pole hypoM	Loss of consciouness + bilateral dystonic arm posturing + oral automatisms	NA	NA
6	Normal	Normal	Left central activity	Left perisylvian Hypo M including anterior temporal gyrus + temporal pole + insula	Bilateral tonic posturing of arms + right head deviation + right arm paresthesias	Left operculo insular thermolesion	la (4 m)
7	Left amygdalar hyperintensity	LT spikes	LT ictal activity	LT pole hypoM + left medial temporal lobe hypoM	Cephalic sensation + dreamy state + language disturbance	Left medial temporal thermolesion	la (5 m)
8	Right HS	RT slow wave activity	RT ictal activity	Right medial temporal hypoM + right anterior temporal neocortical hypoM	Oro alimentary automat. + preserved consciousness + left facial clonus	NA	NA
9	Right HS	RT spikes	RT ictal activity	Right medial temporal hypoM	Verbal automatisms + dysgeusia + loss of consciousness	NA	NA

*HS, hippocampal sclerosis; LT, left temporal; RT, right temporal; hypoM, hypometabolism; ATL, anterior temporal lobectomy; NA, not performed/surgical outcome is expressed as Engel Class*.

Among them, 8 patients presented seizures suggesting a temporal lobe involvement but with clinical features or morphological alterations on brain magnetic resonance imaging (MRI) not typical for medial temporal epilepsy requiring intracranial EEG. For one patient, clinical semiology suggested an involvement of operculo-insular cortex. Three patients underwent surgical resection of the epileptogenic cortex and all had a good surgical outcome (Engel class Ia for all patients with a follow-up duration between 4 and 48 months). For 2 patients, a focal thermolesion using SEEG electrodes was performed, resulting in a dramatic improvement of epilepsy (Engel Ia for both patients with 4 and 5 months of follow-up). For 2 patients, surgery was contraindicated because of the involvement of both temporal lobes during seizures. For 2 patients, surgical resection is planned based on SEEG findings but has not been performed at the time of the present study.

This study, involving human participants, was reviewed and approved by Ethics Committee CPP Lyon Sud EST IV (24/05/2012 N2012-A00516-37). The patients/participants provided their written informed consent to participate in this study.

### 3.2. SEEG Recordings

Intracerebral multi-contact electrodes (5–15 contacts, diameter 0.8 mm, length 2 mm, and 1.5 mm apart) were implanted according to Talairach's stereotactic method (3). Electrode location was verified with post-implantation MRI. Prolonged extra-operative recordings were performed to capture each patient's habitual seizures.

SEEG data were acquired with a 256 channel video EEG monitoring system, Micromed video EEG acquisition system (SD LTM express, Micromed, Treviso, Italy), using the following parameters: sampling rate 256 Hz, high-pass filter 0.15 Hz, low-pass filter 200 Hz, notch filter at 50 Hz.

The median number of bipolar contacts recorded per patient was 101 (range 64–130). The main cerebral structures targeted by intracranial electrodes for each patient are listed in the Supplementary Table 1. As a whole, for all patients, the medial temporal lobe (anterior hippocampus, posterior hippocampus, amygdala, entorhinal cortex), the temporal lateral neocortex and the insular cortex were always targeted. Depending on the electroclinical findings of each patient, frontal lobe, parietal lobe, and occipital cortex were targeted.

For 9 patients, all available seizures were extracted, forming a dataset composed of a total of 27 seizures. For 6 patients, 3 seizures were analyzed. For 2 patients, 4 seizures were analyzed and for 1 patient, a single seizure was available for analysis. For each seizure, we extracted signals for at least 1 min before the onset of the seizure and the whole course of the seizure.

The duration of the seizure was largely heterogeneous both at the inter-individual and intra-individual level. The median length of the seizures across patients was 96 s (range 18–337).

### 3.3. SEEG Signal Analysis

SEEG signals are considered in bipolar derivations; hence each signal is referenced to its closest neighbor. A high-pass filter, with a cut-off frequency equal to 20 Hz at -3 dB was applied on SEEG signals in order to highlight the high-frequency activity typical of seizure activity, particularly at seizure onset (33).

For each seizure, we computed the FC matrices during the pre-seizure period and the course of the whole seizure using a classical connectivity measure, the PLV (24, 25). Briefly, the PLV quantifies the synchronization in phase between two signal. The phase of each signal is computed by the Hilbert transform. The PLV is the time average of the relative phase difference. To compute the FC matrices, the SEEG signals were windowed with 4 s sliding windows moving by steps of 1 s. For each 4 s window, the PLV between all pairs of bipolar contacts was computed to produce the overall network at each time step.

Finally, the BTND method is applied to the set of several seizures for each patient, to decompose all seizures in several subgraphs, activating through time.

### 3.4. Comparison of Network Dynamics Estimated Through Conventional Visual Analysis and the BTND Method

Each seizure was pragmatically segmented in three main periods defined by visual analysis: seizure-onset, seizure propagation, and seizure ending. Visual analysis of the seizures was performed by an expert in clinical SEEG interpretation (JJ). Seizure onset corresponded to the time period with a dramatic change of SEEG signals with either low-voltage fast activity (typically above 20 Hz) or rhythmic spikes in a subset of electrode contacts. Seizure propagation corresponded to an extended time period where ictal SEEG discharge spread to several brain structures either locally or remotely from the seizure onset zone. The recruitment of these regions in the propagation zone can happen either by independent activation of the single areas or by activating multiple areas at the same time. Lastly, a seizure was supposed to have ended when the activation across brain structures was mostly synchronous (typically synchronous spikes) and stable in time and resolved ultimately.

For each time period, we determined the electrode contacts that were involved in the ictal wave with a conventional visual inspection. The electrode contacts were then pooled in several anatomical predefined subregions.

For each patient, the output of the BTND method provided the temporal profile of activation of several common subgraphs of the whole network during each seizure. The list of activated subgraphs at each time period of the seizure was then collected. Each subgraph included several contacts with strong functional connectivity. At each time period, we determined which anatomical subregions were connected based on the activated subgraphs.

Lastly, a qualitative comparison between the set of activated structures determined by visual analysis and the BTND method was performed for each seizure.

### 3.5. Results for the Real Dataset of Epileptic Patients

The overall functional connectivity organization was extracted in all 27 seizures using the BTND method. This means that the seizures (from 1 to 4) of one patient are processed together according to BTND. Table 2 provides the qualitative comparison between the set of activated structures between visual analysis and the BTND method for patients 1 and 2. Also, Tables 3, 4 show the same qualitative comparison for, respectively, patients 2–4 and patients 5–9.

**Table 2:** Qualitative comparison between the set of activated structures determined by visual analysis and the BTND method for the patient 1 and 2.

		**Seizure onset**	**Seizure propagation**	**Seizure ending**
Patient 1	Seiz. 1	**Clinical**:L ANT HIPPOC + L POST HIPPOC **Method**: subg3 + (subg1)	**Clinical**: L ANT HIPPOC + L POST HIPPOC + L TEMP POLE **Method**: subg3 + subg2 + (subg1)	**Clinical**: L ANT HIPPOC + L POST HIPPOC + L TEMP POLE + L ANT TEMP NEOCORTEX **Method**: subg4 + subg5 + subg6 + (subg1)
	Seiz. 2	**Clinical**:L ANT HIPPOC + L POST HIPPOC **Method**: subg3 + (subg1)	**Clinical**: L ANT HIPPOC + L POST HIPPOC + L TEMP POLE **Method**: subg3 + subg2 + (subg1)	**Clinical**: L ANT HIPPOC + L POST HIPPOC + L TEMP POLE + L ANT TEMP NEOCORTEX **Method**: subg4 + subg5 + subg6 + (subg1)
	Seiz. 3	**Clinical**:L ANT HIPPOC + L POST HIPPOC **Method**: subg3 + (subg1)	**Clinical**: L ANT HIPPOC + L POST HIPPOC + L TEMP POLE **Method**: subg3 + subg2 + (subg1)	**Clinical**: L ANT HIPPOC + L POST HIPPOC + L TEMP POLE + L ANT TEMP NEOCORTEX **Method**: subg4 + subg5 + subg6 + (subg1)
	subgraphs	**subg1**: L ANT HIPPOC + L AMYG, **subg2**: L ANT HIPPOC + L POST HIPPOC + L TEMPORAL POLE + L AMYG, **subg3**: L ANT HIPPOC + L POST HIPPOC + L POST TEMPORAL NEOCORTEX + L AMYG, **subg4**: L POST TEMPORAL NEOCORTEX + L TEMPORAL POLE + L ANT HIPPOC + L POST HIPPOC **subg5**: L ANT TEMPORAL NEOCORTEX + L TEMP POLE, **subg6**: L TEMPORAL POLE + L ANT TEMPORAL NEOCORTEX.		
Patient 2	Seiz. 1	**Clinical**: L ANT HIPPOC + L POST HIPPOC **Method**: subg1 + (subg7)	**Clinical**: L ANT HIPPOC + L POST HIPPOC + L ANT TEMPORAL NEOCORTEX + L ORBITO FRONTAL NEOCORTEX **Method**: subg2 + (subg7)	**Clinical**: R ANT HIPPOC + R POST HIPPOC + R AMYG **Method**:subg3 + subg4 + subg5 + subg6 + (subg7)
	Seiz. 2	**Clinical**: R ANT HIPPOC + R POST HIPPOC + R AMYG + R ENTORHINAL CORTEX **Method**: subg3 + subg4 + (subg7)	**Clinical**: R ANT HIPPOC + R POST HIPPOC + R AMYG + R ENTORHINAL CORTEX + R ANT TEMPORAL NEOCORTEX **Method**: subg5 + (subg7)	**Clinical**: L ANT HIPPOC + L POST HIPPOC **Method**: subg1 + subg2 + subg6 + (subg7)
	Seiz. 3	**Clinical**: R ANT HIPPOC + R POST HIPPOC + R AMYG + R ENTORHINAL CORTEX **Method**: subg3 + subg4 + (subg7)	**Clinical**: R ANT HIPPOC + R POST HIPPOC + R AMYG + R ENTORHINAL CORTEX + R ANT TEMPORAL NEOCORTEX **Method**: subg5 + (subg7)	**Clinical**: L ANT HIPPOC + L POST HIPPOC **Method**: subg1 + subg5 + subg6 + (subg7)
	subgraphs	**subg1**: L ANT HIPPOC + L POST HIPPOC, **subg2**: L ANT HIPPOC + L POST HIPPOC + L ENTORHINAL CORTEX + L ORBITO FRONTAL CORTEX, **subg3**: R ANT HIPPOC + R POST HIPOC + R AMYG + R ENTORHINAL CORTEX, **subg4**: R ANT HIPPOC + R POST HIPOC + R AMYG + R ENTORHINAL CORTEX, **subg5**: R ANT HIPPOC + R POST HIPPOC + R ENTORHINAL CORTEX + R ORBITO FRONTAL CORTEX + R ANT TEMPORAL NEORTEX, **subg6**: R ANT TEMPORAL NEOCORTEX + R POST TEMPORAL NEOCORTEX, **subg7**: L ANT TEMPORAL NEOCORTEX		
**Legend**; subg: subgraph, Seiz.: seizure, L: left, R: right, ANT HIPPOC: anterior hippocampus, POST HIPPOC: posterior hippocampus, ANT TEMPORAL NEOCORTEX: anterior temporal neocortex, POST TEMPORAL NEOCORTEX: posterior temporal neocortex, AMYG: amygdala.				

**Table 3:** Qualitative comparison between the set of activated structures determined by visual analysis and the Brain-wide Time-varying Network Decomposition (BTND) method for the patients 3, 4, and 5 (see legend Table 2).

		**Seizure onset**	**Seizure propagation**	**Seizure ending**
Patient 3	Seiz. 1	**Clinical**: R ANT HIPPOC **Method**: (subg2)	**Clinical**: R ANT HIPPOC + R POST HIPPOC + R ENT CX **Method**: subg1	**Clinical**: R ANT HIPPOC + R POST HIPPOC + R ENT CX + R TEMPORAL LATERAL + R OCCIPITAL CX + R PARIETAL CX + R POST CENTRAL OPERC **Method**: subg3 + subg4 + subg5 + subg6
	Seiz. 2	**Clinical**: R ANT HIPPOC + R POST HIPPOC **Method**: (subg2) + subg3 + subg5	**Clinical**: R ANT HIPPOC + R POST HIPPOC + R ENT CX **Method**: subg1	**Clinical**: R ANT HIPPOC + R POST HIPPOC + R ENT CX + R TEMPORAL LATERAL + R OCCIPITAL CX + R PARIETAL CX + R POST CENTRAL OPERC **Method**: subg3 + subg4 + subg5 + subg6
	Seiz. 3	**Clinical**: R ANT HIPPOC + R POST HIPPOC **Method**: (subg2) + subg3 + subg5	**Clinical**: R ANT HIPPOC + R POST HIPPOC + R ENT CX + R TEMPORAL LATERAL + R OCCIPITAL CX + R PARIETAL CX + R POST CENTRAL OPERC **Method**: subg1 + (subg2) + subg4 + subg6	**Clinical**: R ANT HIPPOC + R POST HIPPOC + R ENT CX + R TEMPORAL LATERAL + R OCCIPITAL CX + R PARIETAL CX + R POST CENTRAL OPERC **Method**: (subg2) + subg4
	subgraphs	**subg1**: R ANT HIPPOC + R POST HIPPOC + R AMYG + R ENT CX, **subg2**: R ANT HIPPOC, **subg3**: R PARIETAL CX + R OCCIPITAL CX, **subg4**: R TEMP LATERAL, **subg5**: R TEMP LATERAL + R POST CENTRAL OPERC, **subg6**: R TEMP LATERAL,		
Patient 4	Seiz. 1	**Clinical**: L ANT HIPPOC + L POST HIPPOC + L TEMP POLE **Method**: subg2 + (subg1)	**Clinical**: L ANT HIPPOC + L POST HIPPOC + L TEMP POLE + L POST TEMP NEOCORTEX + R AMYG + R ANT HIPPOC **Method**: subg3 + subg4 + subg5	**Clinical**:L ANT HIPPOC + L POST HIPPOC + L TEMP POLE + L POST TEMP NEOCORTEX + R AMYG + R ANT HIPPOC **Method**: subg4 + subg5 + (subg6)
	Seiz. 2	**Clinical**: L ANT HIPPOC + L POST HIPPOC + L TEMP POLE **Method**: subg2 + (subg1)	**Clinical**: L ANT HIPPOC + L POST HIPPOC + L TEMP POLE + L POST TEMP NEOCORTEX + R AMYG + R ANT HIPPOC **Method**: subg3 + subg4 + subg5	**Clinical**: L ANT HIPPOC + L POST HIPPOC + L TEMP POLE + L POST TEMP NEOCORTEX + R AMYG + R ANT HIPPOC **Method**: subg4 + subg5 + (subg6)
	Seiz. 3	**Clinical**: L ANT HIPPOC + L POST HIPPOC + L TEMP POLE **Method**: subg2 + (subg1)	**Clinical**: L ANT HIPPOC + L POST HIPPOC + L TEMP POLE + L POST TEMP NEOCORTEX + R AMYG + R ANT HIPPOC **Method**: subg3 + subg4 + subg5	**Clinical**: L ANT HIPPOC + L POST HIPPOC + L TEMP POLE + L POST TEMP NEOCORTEX + R AMYG + R ANT HIPPOC **Method**: subg4 + subg5 + (subg6)
	Seiz. 4	**Clinical**: L ANT HIPPOC + L POST HIPPOC + L TEMP POLE **Method**: subg2 + (subg1)	**Clinical**: L ANT HIPPOC + L POST HIPPOC + L TEMP POLE + L POST TEMP NEOCORTEX + R AMYG + R ANT HIPPOC **Method**: subg3 + subg4 + subg5	**Clinical**: L ANT HIPPOC + L POST HIPPOC + L TEMP POLE + L POST TEMP NEOCORTEX + R AMYG + R ANT HIPPOC **Method**: subg4 + subg5 + (subg6)
	subgraphs	**subg1**: L ANT TEMP NEOCORTEX + R ANT HIPPOC, **subg2**: L ANT HIPPOC + L POST HIPPOC, **subg3**: L POST TEMPORAL NEOCORTEX + L AMYGDALA + L ANTERIOR CINGULATE, **subg4**: R AMYGD + R ANT HIPPOC + L ANT HIPPOC + L AMYG, **subg5**: R AMYGD + R ANT HIPPOC **subg6**: L TEMP LATERAL + L TEMP POST		
Patient 5	Seiz. 1	**Clinical**: R ANT HIPPOC + R POST HIPPOC + R TEMP POLE + R AMYG **Method**: subg3 + (subg1)	**Clinical**: R ANT HIPPOC + R POST HIPPOC + R TEMP POLE + R AMYG **Method**: subg3 + subg2 + (subg1)	**Clinical**:R ANT HIPPOC + R POST HIPPOC + R TEMP POLE + R AMYG **Method**: subg3 + (subg4) + (subg1)
	Seiz. 2	**Clinical**: R ANT HIPPOC + R POST HIPPOC + R TEMP POLE + R AMYG **Method**: subg3 + (subg1)	**Clinical**:R ANT HIPPOC + R POST HIPPOC + R TEMP POLE + R AMYG + R POST TEMP NEOCORTEX **Method**: subg3 + subg2 + (subg1)	**Clinical**: R ANT HIPPOC + R POST HIPPOC + R TEMP POLE + R AMYG + R POST TEMP NEOCORTEX **Method**: subg3 + (subg4) + (subg1)
	Seiz. 3	**Clinical**: R ANT HIPPOC + R POST HIPPOC + R TEMP POLE + R AMYG **Method**: subg3 + (subg1)	**Clinical**: R ANT HIPPOC + R POST HIPPOC + R TEMP POLE + R AMYG + R POST TEMP NEOCORTEX + R ORBITO FRONTAL CX **Method**: subg3 + subg2 + (subg1)	**Clinical**: R ANT HIPPOC + R POST HIPPOC + R TEMP POLE + R AMYG + R POST TEMP NEOCORTEX R ORBITO FRONTAL CX **Method**: subg3 + (subg4) + (subg1)
	Seiz. 4	**Clinical**: R ANT HIPPOC + R POST HIPPOC + R TEMP POLE + R AMYG **Method**: subg3 + (subg1)	**Clinical**: R ANT HIPPOC + R POST HIPPOC + R TEMP POLE + R AMYG + R POST TEMP NEOCORTEX **Method**: subg3 + subg2+ (subg1)	**Clinical**: R ANT HIPPOC + R POST HIPPOC + R TEMP POLE + R AMYG + R POST TEMP NEOCORTEX **Method**: subg3 + (subg4) + (subg1)
	subgraphs	**subg1**: R LAT TEMP NEOCORTEX + R LAT FRONTAL CORTEX, **subg2**: R ANT HIPPOC + R POST HIPPOC + R AMY + R TEMPORAL POLE, **subg3**: R ANT HIPPOC + R POST HIPPOC **subg4**: R POST HIPPOC + R ORBITO FRONTAL CX + R TEMPORAL POST NEOCORTEX		

**Table 4:** Qualitative comparison between the set of activated structures determined by visual analysis and the Brain-wide Time-varying Network Decomposition (BTND) method for the patients 6, 7, 8, and 9 (see legend Table 2).

		**Seizure onset**	**Seizure propagation**	**Seizure ending**
Patient 6	Seiz. 1	**Clinical**: L PRECENTRAL OPERCULUM + L POST CENTRAL OPERCULUM + L FRONTAL CORTEX **Method**: subg2 + subg3 + subg4 + (subg1) (subg5)	**Clinical**: L PRECENTRAL OPERCULUM + L POST CENTRAL OPERCULUM + L FRONTAL CORTEX **Method**: subg2 + subg3 + subg4 + (subg1) (subg5)	**Clinical**: L PRECENTRAL OPERCULUM + L POST CENTRAL OPERCULUM + L FRONTAL CORTEX **Method**: subg4 + (subg1) (subg5)
	Seiz. 2	**Clinical**: L PRECENTRAL OPERCULUM + L POST CENTRAL OPERCULUM + L FRONTAL CORTEX **Method**: subg2 + subg3 + subg4 + (subg1) (subg5)	**Clinical**: L PRECENTRAL OPERCULUM + L POST CENTRAL OPERCULUM + L FRONTAL CORTEX **Method**: subg2 + subg3 + subg4 + (subg1) (subg5)	**Clinical**: L PRECENTRAL OPERCULUM + L POST CENTRAL OPERCULUM + L FRONTAL CORTEX **Method**: subg4 + (subg1) (subg5)
	Seiz. 3	**Clinical**: L PRECENTRAL OPERCULUM + L POST CENTRAL OPERCULUM + L FRONTAL CORTEX **Method**: subg2 + subg3 + subg4 + (subg1) (subg5)	**Clinical**: L PRECENTRAL OPERCULUM + L POST CENTRAL OPERCULUM + L FRONTAL CORTEX **Method**: subg2 + subg3 + subg4 + (subg1) (subg5)	**Clinical**: L PRECENTRAL OPERCULUM + L POST CENTRAL OPERCULUM + L FRONTAL CORTEX **Method**: subg4 + (subg1) (subg5)
	subgraphs	**subg1**: L PRECENTRAL OPERCULUM + L POST CENTRAL OPERCULUM + L FRONTAL CORTEX, **subg2**: L PRECENTRAL OPERCULUM + L POST CENTRAL OPERCULUM + L FRONTAL CORTEX + L PARIETAL CORTEX, **subg3**: L POST CENTRAL OPERCULUM + L PARIETAL CORTEX, **subg4**: L TEMPORAL LOBE + L POST PARIETAL CORTEX		
Patient 7	Seiz. 1	**Clinical**: L ANT HIPPOC + L POST HIPPOC + L AMYGD **Method**: subg2 + (subg1)	**Clinical**: L ANT HIPPOC + L POST HIPPOC + L AMYGD + L ANT TEMPORAL NEOCORTEX **Method**: subg3 (+subg1)	**Clinical**: L ANT HIPPOC + L POST HIPPOC + L AMYGD + L ANT TEMPORAL NEOCORTEX **Method**: subg4 + subg5 + subg6 (+subg1)
	subgraphs	**subg1**: L ANT TEMPORAL NEOCORTEX, **subg2**: L ANT HIPPOC + L POST HIPPOC + L AMYGD, **subg3**: L ANT HIPPOC + L POST HIPPOC + L AMYGD, **subg4**: L ANT TEMPORAL NEOCORTEX, **subg5**: L ANT TEMPORAL NEOCORTEX, **subg6**: L ANT HIPPOC + L POST HIPPOC + L AMYGD.		
Patient 8	Seiz. 1	**Clinical**: R ANT HIPPOC + R POST HIPPOC + R AMYG + R ENTORINAL CORTEX + R TEMP POLE **Method**: subg3 + subg6 + (subg1) + (subg2)	**Clinical**: R ANT HIPPOC + R POST HIPPOC + R AMYG + R ENTORINAL CORTEX + R TEMP POLE **Method**: subg3 + subg4 + (subg1)	**Clinical**: R ANT HIPPOC + R POST HIPPOC + R AMYG + R ENTORINAL CORTEX + R TEMP POLE **Method**: subg1 + subg4 + subg5 + (subg2)
	Seiz. 2	**Clinical**: R ANT HIPPOC + R POST HIPPOC + R AMYG + R ENTORINAL CORTEX + R TEMP POLE **Method**: subg3 + subg6 + (subg2)	**Clinical**: R ANT HIPPOC + R POST HIPPOC + R AMYG + R ENTORINAL CORTEX + R TEMP POLE **Method**: subg5 + subg6 + (subg2)	**Clinical**: R ANT HIPPOC + R POST HIPPOC + R AMYG + R ENTORINAL CORTEX + R TEMP POLE + R ANT TEMPORAL NEOCORTEX **Method**: subg1 + subg4 + subg5 + (subg2)
	Seiz. 3	**Clinical**: R ANT HIPPOC + R POST HIPPOC + R AMYG + R ENTORINAL CORTEX + R TEMP POLE **Method**: subg3 + subg6 + (subg2)	**Clinical**: R ANT HIPPOC + R POST HIPPOC + R AMYG + R ENTORINAL CORTEX + R TEMP POLE **Method**: subg5 + subg6 + (subg2)	**Clinical**: R ANT HIPPOC + R POST HIPPOC + R AMYG + R ENTORINAL CORTEX + R TEMP POLE + R ANT TEMPORAL NEOCORTEX **Method**: subg3 + subg6 + (subg1) + (subg2)
	subgraphs	**subg1**: R ANT TEMPORAL NEOCORTEX + R POST TEMPORAL NEOCORTEX, **subg2**: R POST HIPPOC + R ANT TEMPORAL NEOCORTEX + R POST TEMPORAL NEOCORTEX, **subg3**: R ANT HIPPOC + R AMYG, **subg4**: R ENTORHINAL CORTEX + R TEMPORAL POLE + R ANT TEMPORAL NEOCORTEX, **subg5**: R ANT HIPPOC + R POST HIPPOC + R AMYG, **subg6**: R ANT HIPPOC + R AMYG		
Patient 9	Seiz. 1	**Clinical**: R ANT HIPPOC + R POST HIPPOC + R AMYG + R ENTORINAL CORTEX + R TEMP POLE **Method**: subg2 + (subg1)	**Clinical**: L ANT HIPPOC + L TEMP POLE **Method**: subg2 + subg3 + (subg1)	**Clinical**: R ANT HIPPOC + R POST HIPPOC + R AMYG + R ENTORINAL CORTEX + R TEMP POLE + L TEMP POLE + R ANT TEMPORAL NEOCORTEX L ANT HIPPOC **Method**: subg3 + subg4 + subg5 + subg6
	Seiz. 2	**Clinical**: L ANT HIPPOC + L TEMP POLE **Method**: subg3 + (subg5) + (subg1)	**Clinical**: R ANT HIPPOC + R POST HIPPOC + R AMYG + R ENTORINAL CORTEX + R TEMP POLE **Method**: subg2 + subg3 + (subg5) + (subg1)	**Clinical**: R ANT HIPPOC + R POST HIPPOC + R AMYG + R ENTORINAL CORTEX + R TEMP POLE + L TEMP POLE + R ANT TEMPORAL NEOCORTEX L ANT HIPPOC **Method**: subg4 + subg5 + (subg1)
	Seiz. 2	**Clinical**: L ANT HIPPOC + L TEMP POLE **Method**: subg3 + (subg5) + (subg1)	**Clinical**: R ANT HIPPOC + R POST HIPPOC + R AMYG + R ENTORINAL CORTEX + R TEMP POLE **Method**: subg2 + subg3 + (subg5) + (subg1)	**Clinical**: R ANT HIPPOC + R POST HIPPOC + R AMYG + R ENTORINAL CORTEX + R TEMP POLE + L TEMP POLE + R ANT TEMPORAL NEOCORTEX L ANT HIPPOC **Method**: subg4 + subg5 + (subg6)
	subgraphs	**subg1**: L POST TEMPORAL NEOCORTEX + L ANT TEMP NEOCORTEX + R ANT HIPPOC + R LAT TEMPORAL NEOCORTEX, **subg2**: L ANT HIPPOC + L ANT TEMPORAL NEOCORTEX + L POST TEMPORAL NEOCORTEX, **subg3**: R ANT HIPPOC + R POST HIPPOC + R ENTORHINAL CX + R AMYG, **subg4**: L ANT HIPPOC + L ANT TEMPORAL NEOCORTEX, **subg5**: + L POST TEMPORAL NEOCORTEX + L ANT TEMP NEOCORTEX + R ANT HIPPOC + R LAT TEMPORAL NEOCORTEX, **subg6**: R ANT TEMPORAL NEOCORTEX + R POST TEMPORAL NEOCORTEX		

Using this method, we found, in the case of 6 patients, that 6 distinct functional subgraphs characterized the organization of seizures; 7 subgraphs for 1 patient; 5 subgraphs for another one; and only 4 subgraphs characterized seizures for the last patient. However, for each patient, some subgraphs were more strongly activated before seizure onset or were continuously activated before seizure onset and remained active during the course of the seizures. Those subgraphs were considered as non-specific subgraphs for the ictal events. For 5 patients, 2 subgraphs were non-specific while for 4 patients a single subgraph was non-specific.

At seizure onset, a single subgraph was activated for 1 patient, two subgraphs were activated for 4 patients, three subgraphs were activated for 3 patients, and 5 subgraphs were activated for 1 patient. For 24 seizures in 6 patients, the seizure onset determined by visual analysis overlapped closely with the network disclosed by the BTND method. For those patients, the cortical regions underlying the seizure-onset zone determined through visual analysis were included in the seizure-onset subgraphs. However, the seizure-onset subgraph also included other regions with strong functional connectivity not directly outside of the seizure-onset zone. For one of those 6 patients (Pt 2), the seizure involved either the left or the right medial temporal lobe at seizure onset. The seizure-onset subgraph was different for each seizure, and the lateralization of the activated structures was concordant with visual analysis. For 3 seizures in one patient (Pt 9), the seizure-onset subgraph was discordant from the seizure onset-zone. For this patient, the seizure-onset zone involved either right or left medial temporal lobe depending on the seizure. The seizure-onset subgraph for this patient was wrongly lateralized to the right or the left temporal lobe.

During seizure propagation, there was always a close spatial overlap between the activated subgraphs and the brain regions involved at each part of the seizure in all patients. For the 27 seizures in the 9 patients, the brain regions involved during seizure propagation were included in activated subgraphs. However, the congruence between activated subgraphs and regions disclosed by visual analysis was not perfect: a minority of regions were revealed by the BTND method but was not detected by visual analysis.

During seizure ending, a tight spatial overlap was also observed between activated subgraphs and brain regions determined by visual analysis. For 23 seizures, the brain regions involved at seizure ending were included in activated subgraphs. For 4 seizures in 1 patient, visual analysis disclosed more activated regions than the BTND method (Pt 4).

The detailed results are now presented for two cases (Pt 1 and Pt 2).

**CASE 1:**

Pt 1 is a 49 years old male patient. Presurgical non-invasive investigations suggested left temporal lobe epilepsy but some radiological features were considered as atypical for mesial temporal lobe epilepsy syndrome and prompted invasive EEG with SEEG. SEEG targeted several regions within left temporal lobe (anterior hippocampus, posterior hippocampus, amygdala, temporal pole, anterior temporal neocortex, posterior temporal lobe), left orbito frontal cortex, and right temporal lobe (right amygdala, right anterior temporal neocortex).

Three seizures were recorded during SEEG. During the 3 seizures, the initial seizure-onset activity developed in left anterior and posterior hippocampus with secondary involvement of the temporal pole, amygdalar nucleus, and left anterior temporal neocortex at the end of the seizures.

**Figure 2:**
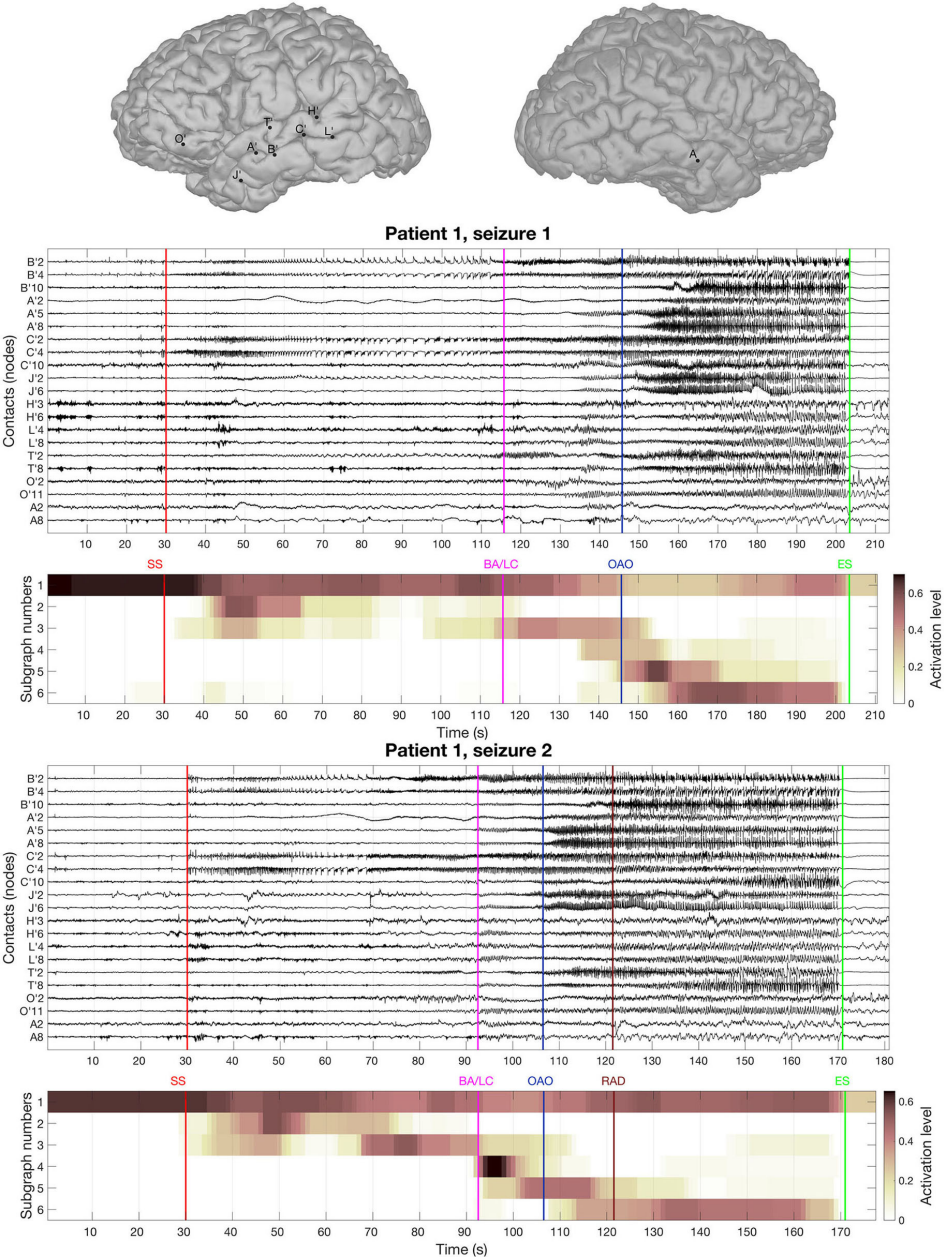
On top is represented the main cerebral structures targeted by intracranial electrodes for the patient 1. Then we picture the recording of two seizures of the patient 1 for selected electrode contacts. Under each seizure, we show the activation level of each subgraph obtained by the BTND decomposition. A subgraph is composed of pairs of contacts with high FC values. The activation level shows the FC dynamic of a subgraph. The main features of ictal semiology are represented by vertical lines, SS, start of the seizure; BA/LC, clinical onset with behavioral arrest and loss of consciousness; OAO, oro alimentary automatisms; RAD, right arm dystonia; SE, end of the seizure.

**Figure 3:**
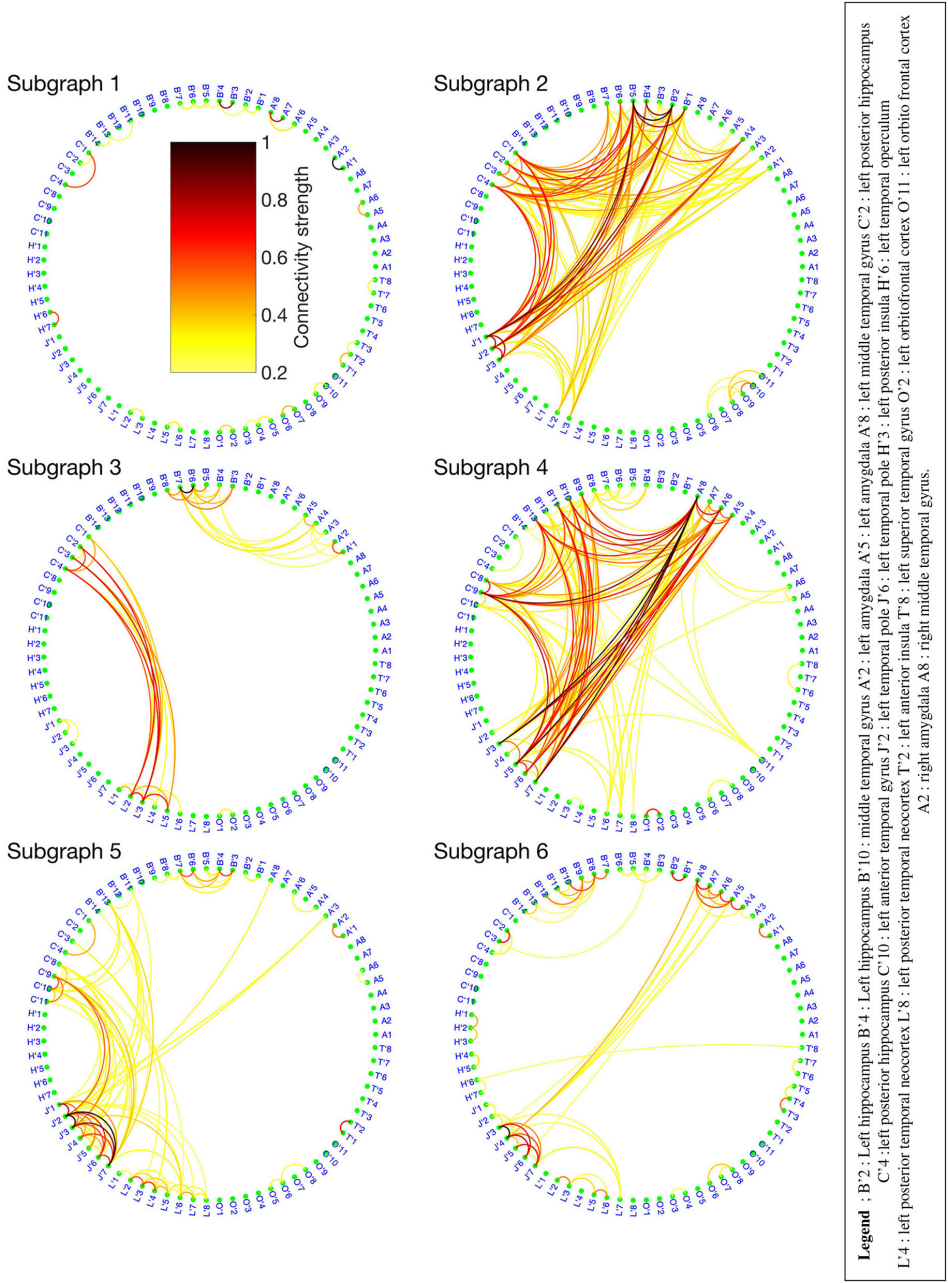
Six functional connectivity (FC) subgraphs revealed by the Brain-wide Time-varying Network Decomposition (BTND) decomposition for the patient 1. The colorbar is the same for each graph. Only the electrode contacts that show one or several connections in at least one subgraph are represented.

The BTND method applied to the three seizures decomposed the connectivity pattern in 6 subgraphs. Figure 2 shows the recording of two seizures (seizure 1 and 2) of the patient 1 for selected electrode contacts. Below each recording, we provide the activation profiles of all subgraphs obtained by the BTND for this specific seizure. On top is represented the main cerebral structures targeted by intracranial electrodes for this patient. Figure 3 shows the 6 FC subgraphs revealed by the BTND.

One subgraph was active before the seizure and during the whole course of the seizures. This subgraph was mostly composed of local connections within temporal lobe (mostly within anterior hippocampus, posterior hippocampus, amygdala). At the seizure onset, during the first seconds of the seizure, there was a reproducible activation of one subgraph, that involved mostly connections between anterior hippocampus and amygdala, posterior hippocampus, and posterior temporal neocortex. A few seconds later, a strong activation of another subgraph was observed that involved mostly connections between medial temporal lobe and temporal pole. During the course of the seizures, there was a consistent activation of three other subgraphs, with a very similar pattern between seizures. Those subgraphs contained mostly connections between anterior lateral temporal neocortex, temporal posterior neocortex, and temporal pole. As a whole, the pattern of activations was very similar for the three seizures and was very consistent with visual analysis of the seizures.

From a clinical point of view, at seizure onset, the patient was asymptomatic and the seizure remained clinically silent for almost 80 s. First clinical symptoms (behavioral arrest and loss of consciousness) occurred more than 1 min after seizure onset during the course propagation. Secondary clinical manifestations included oro alimentary automatisms (seizure 1) and right arm dystonia (seizure 2). Those symptoms occurred while several modules were simultaneously activated.

The patient underwent left anterior lobectomy that resulted in seizure freedom with more than 24 months of follow-up.

**CASE 2:**

Pt 2 is a 37 years old female patient. Presurgical non-invasive investigations suggested that both temporal lobes could trigger habitual epileptic seizures of the patient. Intracranial EEG using SEEG was thus required to evaluate the intrinsic epileptogenicity of each temporal lobe. Intracranial SEEG electrodes targeted mostly both medial and lateral temporal lobes (left and right anterior hippocampus, left and right temporal pole, right amygdala, left and right anterior temporal neocortex, left and right posterior temporal neocortex, left and right insula), but also left and right orbitofrontal cortex.

Three seizures were recorded during SEEG. For the seizure 1, seizure-onset was characterized by a rapid discharge in the left anterior and posterior hippocampus with secondary spread to left anterior lateral temporal and left orbito frontal cortex fast activity and at the end of the seizure propagation to right temporal lobe (right hippocampus and right amygdala). For two seizures (seizures 2 and 3), the initial seizure-onset activity developed in right hippocampus, right amygdala, and right entorhinal cortex with a secondary ictal spread to right anterior temporal cortex and with a propagation to left temporal lobe at seizure ending.

**Figure 4:**
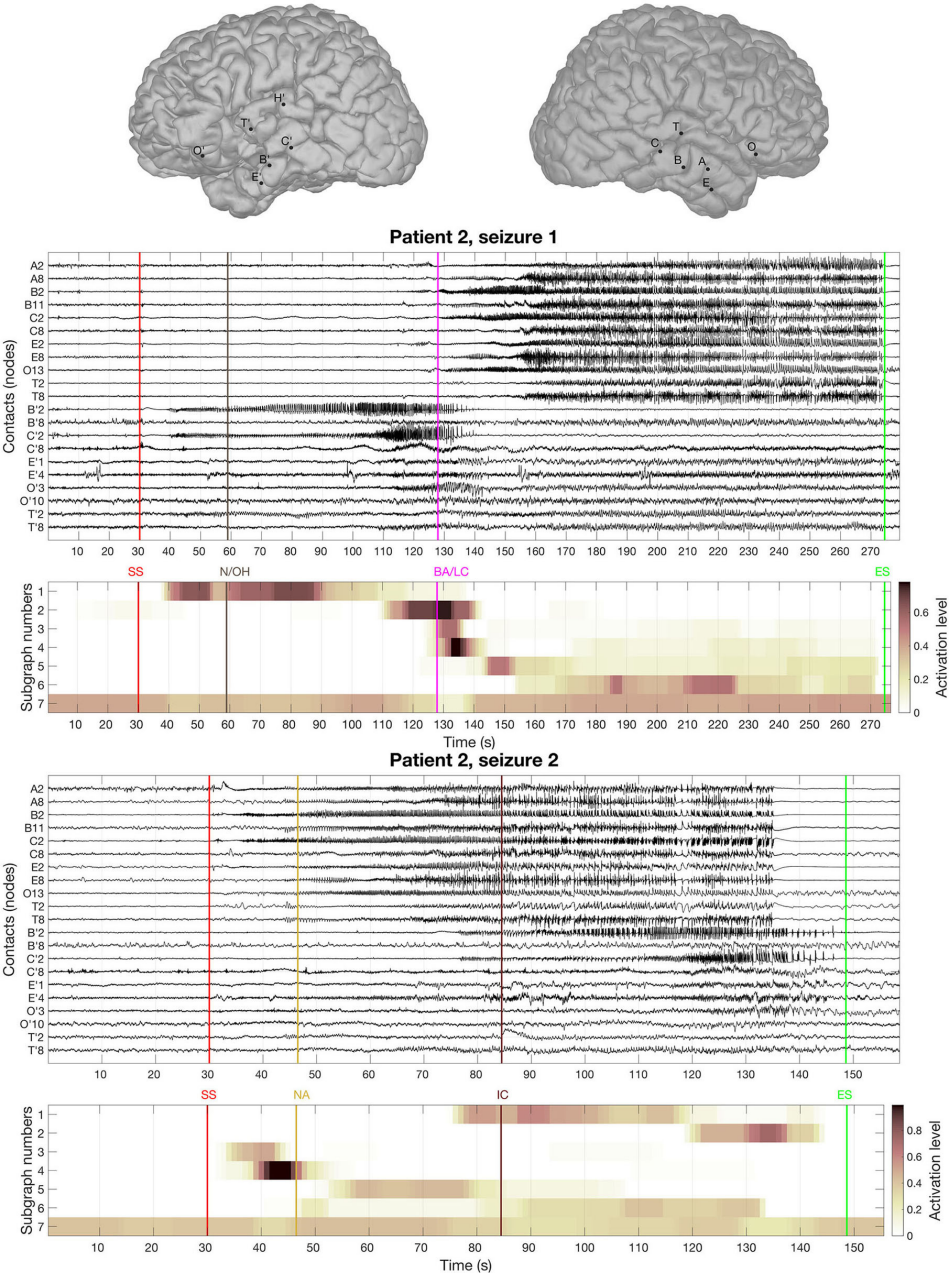
On top is represented the main cerebral structures targeted by intracranial electrodes for patient 2. Then we picture the recording of two seizures of the patient 2 for selected electrode contacts. Under each seizure, we show the activation level of each subgraph obtained by the Brain-wide Time-varying Network Decomposition (BTND) decomposition. A subgraph is composed of pairs of contacts with high functional connectivity (FC) values. The activation level shows the FC dynamic of a subgraph. The main features of ictal semiology are represented by vertical lines, BA/LC, behavioral arrest and loss of consciousness; N/OH, clinical onset with nausea and olfactory hallucinations; NA, nocturnal arousal; IC, ictal confusion.

**Figure 5:**
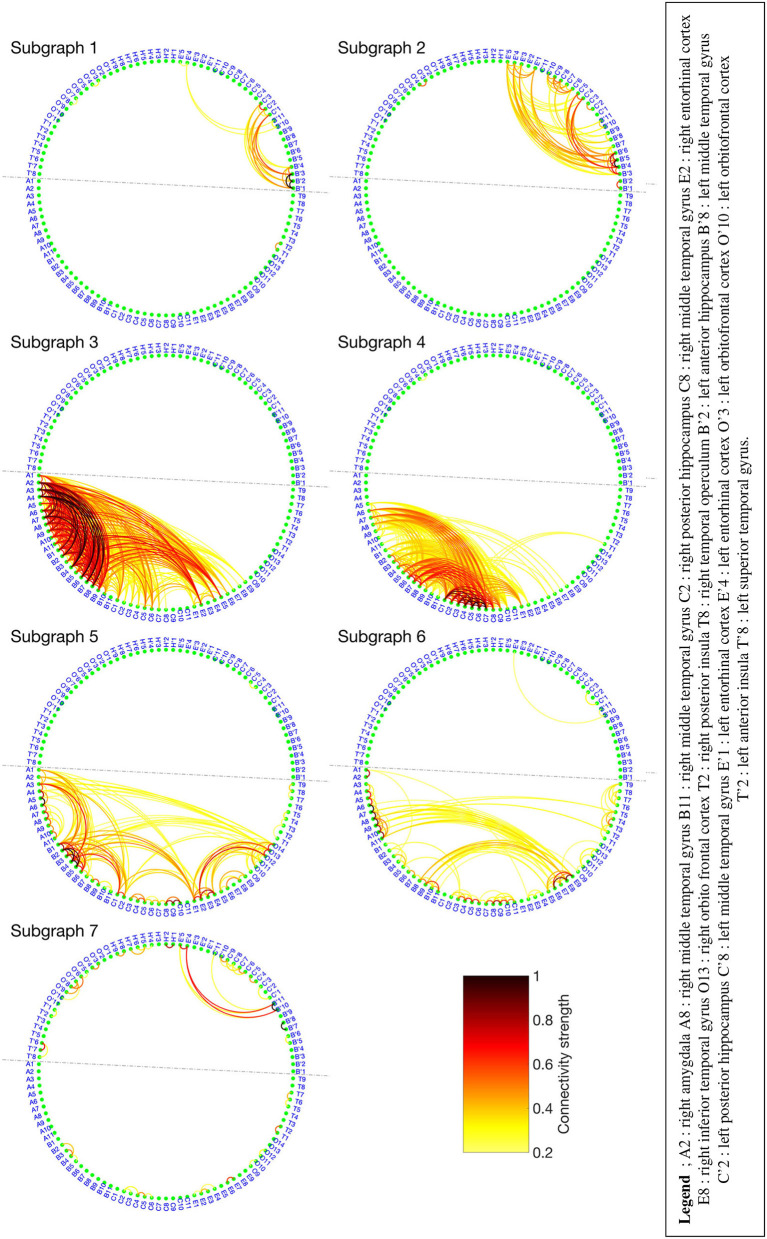
Seven functional connectivity (FC) subgraphs revealed by the Brain-wide Time-varying Network Decomposition (BTND) decomposition for the patient 2. Only the electrode contacts that show one or several connections in at least one subgraph are represented. The dotted line separates the electrode contacts located in the right hemisphere from those located in the left hemisphere.

The BTND method applied to the three seizures decomposed the connectivity pattern in 7 subgraphs. Figure 4 shows the recording of two seizures (seizures 1 and 2) of the Patient 2 for selected electrode contacts. Below each recording, we provide the activation profiles of all subgraphs obtained by the BTND for this specific seizure. On top is represented the main cerebral structures targeted by intracranial electrodes for this patient. Figure 5 shows the 7 FC subgraphs revealed by the BTND.

One subgraph was mostly composed of connections within left medial temporal lobe and left orbitofrontal cortex. Another subgraph was mostly composed of connections within left anterior temporal neocortex. The remaining five subgraphs consisted of regions connecting mostly right medial temporal structures (hippocampus, amygdala, entorhinal cortex) and/or right lateral temporal neocortex. The time course of activation of those subgraphs was closely related to the ictal involvement of both temporal lobes revealed by visual analysis of the seizures: involvement of the left (right) medial temporal lobe was paralleled by an activation of the left (right) subgraphs in a timely fashion, depending on the seizure.

Clinically, ictal semiology for seizure 1 consisted of nausea and olfactory hallucinations with preserved consciousness reported consciously by the patient 30 s after EEG onset followed by behavioral arrest with loss of consciousness more 60 seconds later. Loss of consciousness occurred lately during the course of the seizure when both temporal lobes were involved. At that time, the BTND method showed activation of modules in both temporal lobes. Seizure 2 was a nocturnal seizure with mild clinical semiology, mostly consisting of nocturnal arousal and confusion.

Since SEEG revealed an intrinsic epileptogenicity of both temporal lobes, surgical resection was contraindicated.

## 4. Discussion

This study investigated a new method named BTND to decompose the multi-seizure brain-wide time-varying network obtained by means of FC. The dataset is visualized as FC sub-graphs characterizing the dynamic of all seizures from the same patient. The FC measure used was the PLV, estimated at different time steps of the seizure and applied on large band signal (20–100 Hz). We compared the obtained decomposition of ictal events from 9 patients who have drug-resistant focal epilepsy (observation of a total of 27 seizures) to the visual interpretation from the clinician. Overall, for every patient, results consisting of spatially localized FC subgraphs with stepwise activation were easily interpretable. For 8 of the 9 patients, the decomposition matched with the clinical observation entailing the BTND method as a relevant tool to visualize the multi-seizure brain-wide time-varying network.

### 4.1. Investigating Seizure Dynamics With Brain-Wide Time-Varying Network

It is well-established that the brain is a complex network, with a sophisticated structural connectivity architecture and specific anatomical networks shaping sensory and cognitive processes (34, 35). Functional connectivity measures are a statistical way to investigate the interrelations between brain regions, forming a physiological or pathological brain network (36). When applied on static processes, FC network analysis can provide an instantaneous picture of a stable network. In the field of clinical neuroscience, this proved to be useful for studying stable disease traits like the effects of specific lesions (10) or the effects of drugs on the brain (6, 11, 37). Graph theory-based measures can then provide quantitative tools to explore the overall topology of the network (35). Thus, specific metrics such as modularity, clustering coefficients, or efficiency originating from graph theory (16) were proposed as a useful strategy, for example, to classify patients with Alzheimer disease from standard patients (9) or to explain the effect of physical activity on relations between brain regions (38).

However, static network analysis does not capture one fundamental property of epileptic seizures, the dynamic propagation of the ictal wave. Analysis of time-varying network inferred by dynamic FC measures is a recent topic. Newly emerging dynamic measures that quantify how community organization evolved in time have been proposed in recent years (39). In this context, Kerr et al. (40) shows that simple metrics using the first eigenvector of each FC network lead to the separation of ictal, pre-ictal, or non-ictal events of a recording. A similar metric demonstrated in (22) used to describe the seizure as a succession of states. The strategy can be used to decompose the time axes in states, and then to extract the major FC network of each state (41). Despite producing intelligible results, this strategy hampers the identification of FC subgraphs with interconnected temporal activation, and prevents highlighting some complex relations between brain regions. A complementary approach has been proposed in (23): the authors identify first the main subgraphs of the seizures by modularity optimization (42), and the evolution in time of the main subgraphs leads to a decomposition of the seizure in time states. It should be noticed that this approach is not fundamentally different from the study of static graphs since the subgraphs and their time evolution are determined independently. However, in addition to finding a measure characterizing each FC graph, one must know how to analyze the temporal evolution of the proposed scores. It becomes even more complicated when several modalities are used, as in (43), where several seizures are analyzed for different frequency bands over time.

The present study proposes another strategy, consisting of decomposing all modalities characterizing the dataset simultaneously. Thus, the main advantages of the proposed method are that the analysis pipeline provides both the subgraphs and their temporal activations. The output of the process highlights the main components of the connectivity structure and summarizes a large amount of data with an automatic approach. A similar approach has already been employed in the context of epilepsy in (44) to decompose FC matrices from seizures into FC subgraphs with their respective activation profile. Khambhati et al. (44) demonstrate that inferred FC subgraphs during interictal periods can predict brain regions that generate seizures, and that those subgraphs undergo slower and more coordinated fluctuations during the ictal events compared to interictal states. However, this kind of simultaneous decompositions can produce results that are difficult to interpret. Therefore, for an application different from epilepsy, a sparsity constraint applied on activation profile was shown to enforce intelligibility of the results (45), providing FC subgraphs discriminating the brain network's dynamic in neurodevelopment. Moreover, contrary to our study, the decomposition from (44) does not integrate the different seizures of the same patient to obtain more reproducible FC subgraphs. Tools to decompose several modalities, like tensor decomposition (46–48), were already applied in neuroscience (41, 49, 50) and recently in the context of epilepsy (26). In (26), we proposed a specific tensor decomposition with relevant constraints to encourage the inference of interpretable clusters of FC common to several seizures of the same patient. However, this method is only applicable if all seizures from the same patient have similar durations. In the present study, we developed a new method offering the possibility of decomposing several seizures with different durations from the same patient, which is a more realistic situation in a clinical setting.

### 4.2. Limitations

The BTND method can produce relevant FC, but several limitations have to be addressed. First, the method requires 3 parameters that are directly related to the obtained FC results. We expose a simple procedure to choose each parameter that produces relevant results for each of the 27 seizures. However, the optimal selection of these parameters may be dependent on the FC measure and might be adapted for other clinical applications. Second, for one patient, the BTND method identified a seizure-onset subgraph that was discordant from the seizure onset-zone. For that patient, at seizure-onset, there was a rapid discharge within the seizure-onset zone (without any focal change of synchrony) and a spiking activity within the contralateral hemisphere (accompanied with an increase in synchrony). The increase of FC evaluated by the phase-locking value was wrongly lateralized, emphasizing that the BTND method is dependent on the FC measure used to infer the brain-wide time-varying network. Thus, using another FC measure could be envisaged for this patient. In addition, several studies have already shown that seizure-onset is often marked by a dramatic decrease in seizure connectivity among recorded brain structures. At the same time, synchrony increases progressively during the seizure (51, 52). It might be thus expected that seizure-onset should be characterized by a decrease of activation of subgraphs located within the seizure-onset zone with the BTND method in some patients. It is important to consider that our procedure only highlights functional subgraphs associated with high values of functional connectivity. Thus, it is ideal for showing activations of synchrony in different areas of the brain. However, it is not able to explicitly demonstrate the deactivations that may occur in the brain at the early start of the seizure when SEEG activities in different areas of the brain are suddenly decorrelated. Lastly, direct validation of the method is out of reach since there is no perfect gold standard for the estimation of the connectivity pattern in epileptic patients. We chose to make a correspondence between the propagation patterns disclosed by classical visual interpretation of SEEG signals and the BTND method. Simulation studies generating neural models of epileptic activity with a known connectivity pattern could represent an alternative in future studies.

### 4.3. Clinical Application of the Method

In the present study, we presented an automatic method to describe the connectivity structures of epileptic seizures using an original algorithm with several constraints optimized for that clinical context.

As a whole, we found that the method produces several subgraphs of connections with their activation time course that parallel the patterns of propagation of the seizures closely. In 24/27 seizures, one or two subgraphs overlapped clearly with the seizure onset zone. This suggests that the method can help to localize the seizure-onset zone if there is an increase in synchrony at seizure-onset revealed by FC. This finding confirms several studies evidencing that focal modulations of synchrony help to localize the seizure-onset zone to be surgically resected (5, 53). Moreover, thanks to the sparsity and temporal coherence constraints, the method effortlessly reveals how FC synchrony propagates to the different regions of the brain. As a whole, the method summarizes a vast amount of complex interactions with high readability. We believe that the method is thus highly valuable in clinical studies focusing on connectivity in epileptic patients. For example, this may help to unravel the neural bases of clinical semiology of seizures, which are often related to ictal dysfunction of widespread brain networks involving cortical or subcortical structures. The BTND method provides an exhaustive view of the structure of functional networks at each period of the seizure enabling a fine-grained correlation ictal semiology and network analysis. Lastly, clinical interpretation of SEEG signals is mostly focused on changes of power in large frequency bands (typically high-frequency above 20 Hz at seizure-onset and low band or large band frequency during propagation) revealed by visual inspection. The BTND method enables the detection of changes of synchrony revealed by functional connectivity measures, which are not necessarily paralleled by changes of power. In that respect, we believe that the method may thus contribute to bringing into clinical practice computational measures complementary to a visual interpretation of seizures.

## 5. Conclusion

We present here a novel approach to decompose epileptic seizures in several time-varying subgraphs with high and reproducible functional connectivity values. The method extracts the most significant subgraphs and their corresponding time course of activation. We suggest that this represents a first step to simplify the interpretation of large datasets of functional connectivity for clinical practice. We believe that this will enable further studies investigating the clinical relevance of networks identification for epilepsy surgery.

## Data Availability Statement

The data analyzed in this study is subject to the following licenses/restrictions: no agreement made to make data publicly available. Requests to access these datasets should be directed to Julien Jung, julien.jung@chu-lyon.fr.

## Ethics Statement

The studies involving human participants were reviewed and approved by CPP Lyon Sud EST IV (24/05/2012 N2012-A00516-37). The patients/participants provided their written informed consent to participate in this study. Written informed consent was obtained from the individual(s) for the publication of any potentially identifiable images or data included in this article.

## Author Contributions

GF: concept, method design, data analysis, bibliographic review, writing contributions to the manuscript introduction, materials and methods and discussion, and submission. PB and PG: critical review of the manuscript and scientific supervision (data analysis). JJ: concept, experiment design, scientific supervision (clinic), bibliographic review, writing contributions to the manuscript introduction, application, and discussion. All authors contributed to the article and approved the submitted version.

## Conflict of Interest

The authors declare that the research was conducted in the absence of any commercial or financial relationships that could be construed as a potential conflict of interest.

## Footnotes

^1^https://github.com/FrusqueGaetan/BTND
