# Supplementary material for: Semi-automatic Extraction of Functional Dynamic Networks Describing Patient's Epileptic Seizures
Source: Front Neurol. 2020 Dec 11;11:579725. doi: 10.3389/fneur.2020.579725 (PMC7759641; doi:10.3389/fneur.2020.579725)
Supplement: Supplementary file 1 [file Presentation_1.pdf]

Supplementary Material

1 SUPPLEMENTARY INFORMATIONS ON THE BTND DECOMPOSITION

We remind that formally the BTND is an instance of join nonnegative matrix factorisation [11], [7], [6], [5] and reads :

$$\begin{aligned} \underset{\mathbf{F},\mathbf{V}\{1\},\ldots,\mathbf{V}\{S\}}{\operatorname{argmin}} \quad & \sum_{s=1}^{S} \zeta_s \mid\mid \mathbf{X}\{s\} - \mathbf{F}\mathbf{V}\{s\}^t \mid\mid_F^2 + \lambda S \sum_{k=1}^{K}\sum_{l=1}^{L} \mid F_{lk} \mid, \\ s.t. \quad & C(\mathbf{V}_{:k}\{S\}, \gamma_s, \eta_s) \;\; \forall k \in \{1,...,K\}, \;\; \forall s \in \{1,...,S\}, \\ s.t. \quad & \mathbf{F} \geq 0, \qquad \mathbf{V}\{s\} \geq 0 \;\; \forall s \in \{1,...,S\}, \end{aligned} \tag{S1}$$

With $\mid\mid \mathbf{Y} \mid\mid_F^2 = \sum_i \sum_j Y_{ij}^2$ the classic sum of square function. ζ_s are parameters to balance the importance of each seizure. ζ_s is selected in order to give to each seizure the same importance, we have $\forall s \in \{1,...,S\}$, we have $\forall s \in \{1,...,S\}$, $\zeta_s = \mid\mid \mathbf{X}\{1\} \mid\mid_F^2 \; / \mid\mid \mathbf{X}\{s\} \mid\mid_F^2$. γ_s and η_s are selected to respectively get the sparsity and compactness level according to the duration of the seizure. if the seizure is short we expect to select less non-null time step, on the contrary, if the seizure is long, we authorise to infer longer activated period of time. We have $\forall s \in \{1,...,S\}$, $\gamma_s = T(1)/T(S)\gamma$ and $\eta_s = T(1)/T(S)\eta$.

Then, the method is associated to three hyperparameters : λ and γ respectively controling the sparsity level of the FC subgraphs and the activation profiles, and η regulating the temporal compactness of the activation profiles.

1.1 Initialisation

We warn that the optimisation problem equation (S1) is non convex, thus different initialisation lead to different solution which are local minima of the cost function equation (S1). To overcome this limitation we employ the usual strategy practised for other non-convex machine learning methods (like the algorithm k-means to perform data clustering [4]). Thereby, we compute for different initialisations a locales minimum of the cost function equation (S1) and retain the best result . 20 different initialisation seems sufficient in practice. One considers $\mathbf{F}$ as the left singular component of the concatenation of the matrices $\mathbf{X}\{s\}$; this initialisation was remarked to be a good starting point. We create the other initialisations using random matrices where each element is drawn from a uniform distribution between 0 and 1.

1.2 Parameters selection

For the parameters selection, we fix for all seizures $\eta = 0.2$ empirically. This value seems to be sufficient to obtain a coherent activation profile each time. We suggest a simple procedure to select automatically parameters λ and γ for every patient. First, as both parameters λ and γ are aimed to obtain sparse components, we propose to merge them by fixing $\lambda = \gamma$. It reduces to the selection of only one parameter λ. Then, we remarked that even for different values of K the effect of the regularization level stays similar. Thus, we fix $K = 5$ for the selection of λ. We are looking for a value λ such as the reconstruction rate $a_\lambda = \sum_{s=1}^{S} \zeta_s \mid\mid \mathbf{X}\{s\} - \mathbf{F}\mathbf{V}^t\{s\} \mid\mid_F^2$, where $\mathbf{F}$ and $\mathbf{V}^t\{s\}$ is the obtained decomposition using the paramter λ, is sufficiently high to ensure most of the background information have been eliminated. We specify that a_λ increase with λ since the regularization limit the capacity of the initial matrices $\mathbf{X}\{s\}$ the be reconstructed. Then, the most fidel reconstruction corrrespond to a_0 associated to fully connected and

complex subgraphs. When the regularization is too strong, matrices $\mathbf{F}$ and $\mathbf{V}\{s\}$ are null, then we note the reconstruction rate in this case $a_\infty = \sum_{s=1}^{S} \zeta_s \mid\mid \mathbf{X}\{s\} \mid\mid_F^2$. Then, we select the parameter λ, such that 80% of the information contained in the most faithful reconstruction is eliminated. This is to say with a reconstruction rate of $a_\lambda \approx 0.8(a_\infty - a_0) + a_0$. To find this approximation we compute the solution of the problem equation (S1) with $\lambda = 0$ to $\lambda = 1$ by step of 0.05 (with 20 different initialisations each time). We choose a threshold of 80% because it leads to select only the most activated functional connectivity. For lower percentage, background connection starts to be reconstructed. A higher percentage can produce more sparse subgraphs; however, they become less explicit in term of pathological connections.

To select the number of subgraphs needed, we compute for $K = 3$ to 10 the decomposition with the selected set of parameters. Both an Elbow criterion and visual inspection of the obtained temporal activation and subgraphs are applied to select the best number K.

1.3 Post processing

We specify that the functional subgraphs $\mathbf{F}$ solution of the problem equation (S1), do not necessarily contain values comprised between 0 and 1; this is problematic to interpret them as functional connectivity measure. Moreover, the lack of scaling does not ease the comparison between different subgraphs. In order to promote interpretable results we perform, as a post processing step, the following normalisation imposing $\mathbf{F}$ to contain values comprised between 0 and 1. We call $m_k = max(\mathbf{F}_{:k})$ the maximum value of the subgraph k. The new normalized subgraph simply corresponds to $\bar{\mathbf{F}}_{:k} = \frac{1}{m_k}\mathbf{F}_{:k}$. To keep relevant activation profiles for each seizure, we trasfert them the factor m_k, with $\bar{\mathbf{V}}_{:k}\{s\} = m_k\mathbf{V}_{:k}\{s\}, \forall s \in \{1, ..., S\}$. Since the regularization function used to impose sparsity is a relaxation of the function that explicitely count the number of non-null functional connectivity in each subgraph, some artifact can appear. We propose then to decrease their presence, by only considering for each subgraph $\bar{\mathbf{F}}_{:k}$ the values higher to 0.2.

1.4 Algorithme

Finally, to stay explicit, we solve this problem by alternate a lasso regression to update the matrix $\mathbf{F}$ knowing matrices $\mathbf{V}\{s\}$. We use a projected gradient descent to update each column of the matrix $\mathbf{V}\{s\}$ knowing $\mathbf{F}$. The lasso regression is done using a proximal gradient algorithm [9] with a FISTA acceleration [1]. The least square regression under fussed lasso constraint alternate the use of a projected gradient descent strategy on each column of the matrices $\mathbf{V}\{s\}$ as proposed in [8] or [3]. The projection on the admissible solutions of the fused lasso constraints use an algorithme related to [2], that can be found in this website[1], based on isotonic regressions [10]. We provide the URL[2] for a Github repository with Matlab implementation of the proposed BNTD method.

[1] https://lcondat.github.io/

[2] https://github.com/FrusqueGaetan/BTND

2 SUPPLEMENTARY TABLES

Patient	1	2	3	4	5	6	7	8	9
Number of recorded bipolar contacts	79	116	130	103	101	135	64	85	95
**ANT HIPPOC**	L & R	L & R	R	L & R	R	L	L	R	L & R
**POST HIPPOC**	L	L & R	R	L & R	R	NA	L	R	R
**AMYGDALA**	L & R	R	R	L & R	R	NA	L	R	R
**ENTORHINAL CORTEX**	NA	R	R	NA	R	NA	L	R	R
**TEMPORAL POLE**	L	NA	R	L & R	R	L	L	R	L & R
**ANT TEMPORAL NEOCORTEX**	L & R	L & R	R	L & R	R	L	L	R	L & R
**POST TEMPORAL NEOCORTEX**	L	L & R	R	L	R	NA	L	R	L & R
**INSULA**	L	L & R	R	L	R	L	L	NA	R
**PRECENTRAL OPERCULUM**	NA	NA	R	NA	NA	L	NA	NA	NA
**POST CENTRAL OPERCULUM**	NA	NA	R	L	NA	L	NA	NA	NA
**PARIETAL CORTEX**	NA	NA	R	NA	NA	L	NA	NA	NA
**OCCIPITAL CORTEX**	NA	NA	R	NA	NA	NA	NA	R	NA
**OFC**	L	L & R	NA	NA	R	NA	NA	NA	NA
**ANT CING**	NA	NA	NA	L	R	L	NA	NA	NA
**LAT FRONTAL CORTEX**	NA	NA	NA	NA	R	L	NA	NA	NA

Table S1. List of the main cerebral structures targeted by intracranial electrodes. **Legend**; L: left, R: right, NA: not performed, ANT HIPPOC: anterior hippocampus, POST HIPPOC: posterior hippocampus, OFC: Orbito Frontal Cortex, ANT CING: Anterior Cingulate, LAT FRONTAL: Lateral Frontal Cortex.

REFERENCES

[1]Amir Beck and Marc Teboulle. A fast iterative shrinkage-thresholding algorithm for linear inverse problems. *SIAM journal on imaging sciences*, 2(1):183–202, 2009.

[2]Laurent Condat. A direct algorithm for 1-D total variation denoising. *IEEE Signal Processing Letters*, 20(11):1054–1057, 2013.

[3]Jerome Friedman, Trevor Hastie, Holger Höfling, and Robert Tibshirani. Pathwise coordinate optimization. *The annals of applied statistics*, 1(2):302–332, 2007.

[4]Anil K. Jain. Data clustering: 50 years beyond K-means. *Pattern recognition letters*, 31(8):651–666, 2010.

[5]Xingpeng Jiang, Xiaohua Hu, and Weiwei Xu. Microbiome data representation by joint nonnegative matrix factorization with laplacian regularization. *IEEE/ACM transactions on computational biology and bioinformatics*, 14(2):353–359, 2015.

[6]Hannah Kim, Jaegul Choo, Jingu Kim, Chandan K. Reddy, and Haesun Park. Simultaneous discovery of common and discriminative topics via joint nonnegative matrix factorization. In *Proceedings of the 21th ACM SIGKDD International Conference on Knowledge Discovery and Data Mining*, pages 567–576, 2015.

[7]Preethi Lahoti, Kiran Garimella, and Aristides Gionis. Joint non-negative matrix factorization for learning ideological leaning on twitter. In *Proceedings of the Eleventh ACM International Conference on Web Search and Data Mining*, pages 351–359, 2018.

[8]Julien Mairal, Francis Bach, Jean Ponce, and Guillermo Sapiro. Online learning for matrix factorization and sparse coding. *Journal of Machine Learning Research*, 11(Jan):19–60, 2010.

[9]Neal Parikh and Stephen Boyd. Proximal algorithms. *Foundations and Trends® in Optimization*, 1(3):127–239, 2014.

[10]Ryan J. Tibshirani, Holger Hoefling, and Robert Tibshirani. Nearly-isotonic regression. *Technometrics*, 53(1):54–61, 2011. ISBN: 0040-1706 Publisher: Taylor & Francis.

[11]Hong-Qiang Wang, Chun-Hou Zheng, and Xing-Ming Zhao. j NMFMA: a joint non-negative matrix factorization meta-analysis of transcriptomics data. *Bioinformatics*, 31(4):572–580, 2015.
